# Echocardiographic Predictors of Ventricular Arrhythmias in Patients With Left Ventricular Assist Devices and Implantable Cardioverter‐Defibrillator

**DOI:** 10.1111/jce.16539

**Published:** 2024-12-16

**Authors:** Elena Efimova, Samira Zeynalova, Sandra Eifert, Alexey Dashkevich, Michael Andrew Borger, Anna L. Meyer, Jens Garbade, Angeliki Darma, Kerstin Bode, Arash Arya

**Affiliations:** ^1^ Department of Electrophysiology Leipzig Heart Center Leipzig Germany; ^2^ Institute of Medical Informatics, Statistics and Epidemiology University of Leipzig Leipzig Germany; ^3^ Department of Cardiac Surgery, University Clinic of Cardiac Surgery Leipzig Heart Center Leipzig Germany; ^4^ Department of Cardiac Surgery Heidelberg University Hospital Heidelberg Germany; ^5^ Department of Cardiac Surgery Klinikum Links der Weser Bremen Germany; ^6^ Department of Cardiology, University Hospital Halle Martin‐Luther University Halle‐Wittenberg Halle (Saale) Germany

**Keywords:** echocardiographic predictors, left ventricular assist device, ventricular arrhythmias

## Abstract

**Aim:**

To evaluate the predictive value of preoperative echocardiographic parameters for occurrence of VAs in patients with preexisting ICD undergoing LVAD implantation.

**Methods and Results:**

All consecutive patients (*n* = 264) with previous ICD who underwent LVAD surgery between May 2011 and December 2019 at our institution were included. The patients were predominantly male (89%) with NICM (59%) and a mean age of 59 ± 10 years. All LVADs were continuous flow device (154 HVAD, 21 HeartMate II, and 89 HeartMate 3). A total of 102 (39%) patients had VAs in the first year after LVAD implantation. We compared echocardiographic parameters in patients with and without VAs before LVAD, at 1 month and 1 year after LVAD implantation. Increased pre‐LVEDD ≥ 72 mm predicted the occurrence of VAs after LVAD implantation for ICM patients (HR: 2.9, 95% confidence interval (CI): [1.3–6.6], *p* = 0.012), while a larger pre‐RVEDD ≥ 46 mm was predictive in NICM patients (HR: 2.8, (CI): [1.4–5.9], *p* = 0.004). Moreover, a larger RVEDD at 1 year after LVAD was highly associated with VAs in the first year after LVAD implantation (50 ± 10 vs. 45 ± 8 mm, *p* = 0.001). All patients demonstrated a significant decrease in LVEDD as well as a reduction in severity of mitral and tricuspid regurgitation during 1 year after LVAD implantation, reflecting left ventricular unloading through the LVAD.

**Conclusions:**

Larger left and right ventricular diameters before LVAD predict the occurrence of VAs after LVAD implantation in ICM and NICM patients. Persistent RV remodeling post‐LVAD is also associated with VAs.

**Abbreviations**
ICM: ischemic cardiomyopathy
IVS: interventricular septum
LGE: late gadolinium enhancement
LVEDD: left ventricular end‐diastolic diameter
LVEF: left ventricular ejection fraction
LVESD: left ventricular end‐systolic diameter
MR: mitral regurgitation
MVT: monomorphic ventricular tachycardia
NICM: nonischemic cardiomyopathy
PVT: polymorphic ventricular tachycardia
RVEDD: right ventricular end‐diastolic diameter
RV‐RA gradient: right ventricular‐to‐right atrial pressure difference
SCD: sudden cardiac death
TR: tricuspid regurgitation
TTE scan: transthoracic echocardiographic scan
VAs: ventricular arrhythmias
VF: ventricular fibrillation

## 1 Introduction

Continuous flow left ventricular assist devices (LVADs) have become a well‐recognized therapy option in patients with advanced heart failure as destination therapy [1]. Determining prognostic factors for better outcomes post‐LVAD has become an increasingly pursued research goal.

Ventricular arrhythmias (VAs) occur frequently after LVAD implantation and are considered to be a predictor for adverse outcome in this patient group [2]. Several studies have demonstrated the feasibility and usefulness of echocardiographic parameters for outcome prediction in patients treated with continuous‐flow LVADs [3, 4, 5, 6]. However, no study has reported on the association between echocardiographic predictors and the occurrence of VAs to date. The aim of this study was to assess the predictive value of different echocardiographic parameters for the occurrence of VAs. We hypothesized that a larger ventricular diameter and a lower left ventricular ejection fraction (LV‐EF) before LVAD are associated with the occurrence of VAs after LVAD implantation.

## 2 Methods

### 2.1 Patient's Population

All consecutive patients, who underwent LVAD implantation between May 2011 and December 2019 at our institution with a pre‐existing implantable cardioverter defibrillator (ICD, *n* = 264) were included in this study. Exclusion criteria were the lack of ICD before LVAD surgery and biventricular VAD implantation. The study was conducted in accordance with the local institutional review board and the standards of the University of Leipzig ethics committee. All patients provided written informed consent. All implanted LVADs were continuous flow left ventricular assist devices (154 HVAD [Medtronic, Minneapolis, Minnesota, United States], 21 HeartMate II [Abbott Laboratories, Illinois, United States], and 89 HeartMate 3 [Abbott Laboratories, Illinois, United States]).

After LVAD implantation, patients were followed up in our clinic 1 month after hospital discharge and then at 3‐month intervals, according to our normal post‐LVAD protocol. Additionally, they underwent weekly telephone interviews. During the clinic visits. patients were questioned about their clinical status, checked for the occurrence of VAs by interrogation of their ICD, underwent transthoracic echocardiography (TTE) along with a check of the LVAD pump parameters and a laboratory test. To clarify a possible relationship between the extent of LV unloading and the occurrence of VAs, we assessed the position of the interventricular septum (IVS) on TTE scan.

### 2.2 ICD Settings

Only episodes of either monomorphic ventricular tachycardia (MVT) or polymorphic ventricular tachycardia (PVT) and/or ventricular fibrillation (VF) terminated by appropriate ICD therapy (anti‐tachycardia pacing or shock) were analyzed. All preoperative ICD settings including biventricular pacing in patients with CRT‐D remained unchanged.

### 2.3 Echocardiographic Measurements

Two‐dimensional TTE was performed in a standard manner using Vivid 7 and Vivid 9 (GE Medical Systems, Milwaukee, WI) within 1 month before LVAD implantation, 1 month postoperatively and at 3‐months intervals thereafter. All examinations were performed by trained staff of the echocardiography department of the Leipzig Heart Center.

According to guidelines, the left ventricular (LV) diameter was measured from the parasternal long‐axis view using two‐dimensional M‐mode echocardiography at the papillary muscle level [7]. The ejection fraction was calculated using the Teichholz method because of the limited LV visualisation due to the device‐related artefacts in a four‐chamber view. Valvular regurgitation was qualitatively assessed using color Doppler, vena contracta width as well as proximal iso‐velocity surface area and graded using 3‐point scale (mild = 1, moderate = 2, severe = 3). Right ventricular (RV) chamber size and function were evaluated in a four‐chamber view at the maximum RV dimension during diastole. Right ventricular function was assessed by estimation of tricuspid annular plane systolic excursion (TAPSE). RV systolic pressure was estimated as the RV‐RA gradient derived from the tricuspid regurgitation (TR) profile. Furthermore, the unloading status was estimated indirectly based on the position of IVS: 0‐neutral position, 1‐rightward shift, 2‐leftward shift.

### 2.4 LVAD Settings

The LVAD pump speed setting was performed intraoperatively under echocardiographic guidance, depending on hemodynamic parameters, position of the IVS, and frequency of the aortic valve opening and then adjusted at follow‐up examinations if necessary.

### 2.5 Statistical Analysis

Statistical analysis was performed using SPSS for Windows version 28.0. All data are expressed as mean ± SD for continuous variables and as numbers and ratios for categorical variables. Comparisons between groups were made using the student *t*‐test for continuous and the *χ* ^2^ test for categorical variables. The influence of various echocardiographic parameters on the occurrence of post‐LVAD VAs was assessed by both uni‐ and multivariate binary regression analysis. The end point was the arrhythmia‐free survival, that was defined as time from LVAD implantation until the first VA episode during the first postoperative year. The arrhythmia‐free survival probabilities were estimated using the log‐rank test and represented on the Kaplan Meier plot as well as by the cox regression analysis. Cutoff values for left ventricular end diastolic diameter (LVEDD) and right ventricular end diastolic diameter (RVEDD) were determined by means of the ROC analysis. All probability values reported are two‐sided, and a probability value of *p* < 0.05 was considered statistically significant.

## 3 Results

### 3.1 Baseline Characteristics

**Figure 1:**
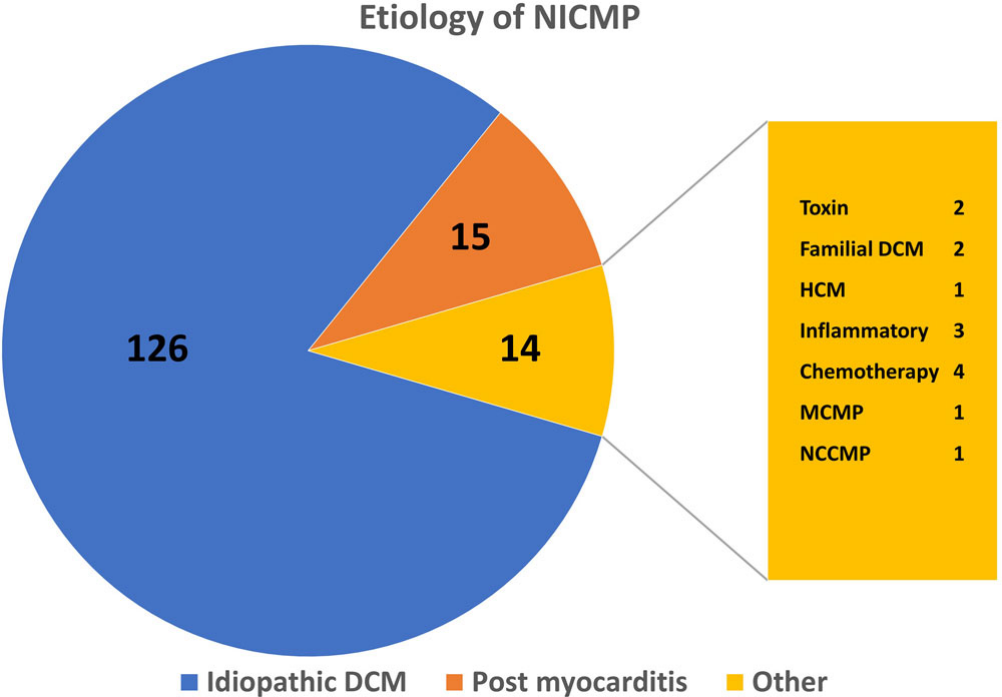
Etiology of NICM in LVAD patients.

Table 1 shows the baseline characteristics of all enrolled patients, divided into two groups with‐ and without VAs after LVAD implantation. The studied cohort was mostly male (89%) with a mean age of 59 ± 10 years and the leading cause of heart failure was nonischemic cardiomyopathy (NICM—59%). Almost all patients (98%) had NYHA IV class of heart failure before LVAD implantation. The NICM group was heterogeneous with a variety of conditions (Figure 1).

**Table 1:** Clinical characteristics of patients with and without ventricular arrhythmias after LVAD implantation.

	All patients	With post LVAD VAs	Without post LVAD VAs	*p*‐value
Patients, n (%)	264	102 (39)*	162 (61)	
Age, years (range)	59 ± 10	59 ± 9	59 ± 10	0.93
Men, n (%)	234 (89)	94 (40)	140 (60)	0.15
NICM, n (%)	155 (59)	61 (39)	94 (61)	0.78
ICM, n (%)	109 (41)	41 (38)	68 (62)	
Atrial fibrillation, n (%)	194 (74)	80 (41)	114 (59)	0.15
Death during 12 month after LVAD, n (%)	55 (21)	18 (33)	37(67)	0.31
Aortic valve surgery before LVAD, n (%)	26 (10)	8 (31)	18 (69)	0.39
Tricuspid valve surgery before LVAD, n (%)	11 (4)	4 (36)	7 (64)	0.87
Mitral valve surgery before LVAD, n (%)	56 (21)	31 (55)	25 (45)	0.008
Bypass surgery before LVAD, n (%)	43 (16)	19 (44)	24 (56)	0.41
ß‐blocker	212 (80)	82 (39)	130 (61)	0.45
Amiodarone	86 (33)	39 (45)	47 (55)	0.19
Sotalol	11 (4)	8 (73)	3 (27)	0.022
ACE inhibitors	109 (41)	38 (35)	71 (65)	0.19
ARBs	39 (15)	13 (33)	26 (67)	0.40
MRAs	206 (78)	80 (39)	126 (61)	0.67
LVAD system, n (%)				
HVAD, n (%)	154 (58)	60(39)	94 (61)	0.61
Heart Mate II, n (%)	21 (8)	10 (48)	11 (52)	
Heart Mate 3, n (%)	89 (34)	32 (36)	57 (64)	
Implanted ICD, n (%)				
Single chamber ICD, n (%)	101 (38)	41 (41)	60 (59)	0.87
Dual chamber ICD, n (%)	39 (15)	15 (38)	24 (62)	
CRT‐D, n (%)	124 (47)	46 (37)	78 (63)	
*Variables are reported as row percentages **Echocardiographic parameters pre‐LVAD**				
LVEF (%)	20 ± 7	21 ± 7	20 ± 7	0.35
LVEDD (mm)	72 ± 11	74 ± 12	71 ± 9	0.02
RVEDD (mm)	47 ± 8	48 ± 9	47 ± 7	0.12
TAPSE (mm)	14 ± 4	14 ± 4	15 ± 4	0.94
RV‐RA pressure gradient (mmHg)	38 ± 13	36 ± 12	39 ± 14	0.05
TR, grade	1.6 ± 0.9	1.6 ± 0.9	1.6 ± 1.0	0.99
MR, grade	1.8 ± 1.0	1.8 ± 1.0	1.9 ± 0.9	0.51
**Echocardiographic parameters post‐LVAD at 1 month**				
LVEF (%)	21 ± 8	20 ± 8	22 ± 8	0.12
LVEDD (mm)	66 ± 12	69 ± 13	65 ± 12	'0.03
RVEDD (mm)	48 ± 8	49 ± 10	47 ± 7	0.05
TAPSE (mm)	12 ± 3	12 ± 3	12 ± 3	0.98
RV‐RA pressure gradient (mmHg)	25 ± 12	26 ± 13	24 ± 11	0.29
TR, grade	1.2 ± 1.0	1.4 ± 1.0	1.1 ± 0.9	0.03
MR, grade	0.9 ± 0.8	0.9 ± 0.8	0.9 ± 0.7	0.98
**Echocardiographic parameters post‐LVAD at 1 year**				
LVEF (%)	22 ± 7	22 ± 6	22 ± 8	0.84
LVEDD (mm)	67 ± 12	67 ± 13	66 ± 11	0.53
RVEDD (mm)	47 ± 9	50 ± 10	45 ± 8	0.001
TAPSE (mm)	12 ± 3	12 ± 3	12 ± 3	0.41
RV‐RA pressure gradient (mmHg)	24 ± 9	24 ± 9	24 ± 9	0.96
TR, grade	1.1 ± 0.9	1.4 ± 1.0	1.0 ± 0.9	0.03
MR, grade	0.9 ± 0.8	0.9 ± 0.8	0.8 ± 0.8	0.25

The mean follow‐up time was 50 ± 33 (range 0–129) months and the mean time from LVAD implantation to the first VA episode in the first year after LVAD was 83 days (range 0–346). The mean time from the last preoperative echocardiographic examination to the time of LVAD implantation was 20 ± 20 days, from the LVAD implantation to the first post‐LVAD echocardiography 27 ± 25 days and then 360 ± 62 days to the last follow‐up echocardiography Table 2.

**Table 2:** Echocardiographic parameters of all LVAD patients (*n* = 264) before LVAD implantation and at 1 year follow‐up.

	Before LVAD	At 1 year after LVAD	*p*‐value
LVEF (%)	20 ± 7	22 ± 7	0.004
LVEDD (mm)	73 ± 11	67 ± 11	< 0.001
RVEDD in 4 chamber view (mm)	47 ± 8	47 ± 9	0.67
TAPSE (mm)	14 ± 3	12 ± 3	< 0.001
RV‐RA pressure gradient (mmHg)	37 ± 13	24 ± 9	< 0.001
TR, grade	1.6 ± 1.0	1.2 ± 0.9	< 0.001
MR, grade	1.9 ± 0.9	0.9 ± 0.8	< 0.001

### 3.2 Echocardiographic Characteristics of Studied Population on LVAD Support

LV unloading during LVAD therapy measured by a decrease of LVEDD and improvement of mitral regurgitation (MR) was demonstrated at the first postoperative scan (73 ± 11 vs. 66 ± 12 mm, *p* < 0.001 for LVEDD and 1.8 ± 1.0 vs. 0.8 ± 0.8, *p* < 0.001 for MR) and remained static without any significant further change throughout the first postoperative year. LV ejection fraction did not demonstrate any changes at the first post‐LVAD control (20 ± 7 vs. 21 ± 8, *p* = 0.2). However, it showed a statistically significant increase at the 1‐year follow‐up (20 ± 7 vs. 22 ± 7, *p* = 0.004). A significant decline of RV‐ right atrial (RA) pressure gradient was observed on the first post‐LVAD echocardiogram (from 37 ± 13 to 24 ± 11 mmHg, *p* < 0.001), but then remained static over the remaining follow‐up period (27 ± 12 vs. 23 ± 10 mmHg, *p* = 0.08). Despite a significant reduction of the estimated RV‐RA gradient pressure (37 ± 14 vs. 24 ± 11 mmHg, *p* < 0.001), the RVEDD did not change significantly in the first postoperative year. Nonetheless, RVEDD showed a trend to reduction at the first postoperative control (49 ± 10 vs. 47 ± 7, *p* = 0.05) as well as at 1 year (48 ± 9 vs. 47 ± 9 mm, *p* = 0.06). We found a small but statistically significant reduction of TAPSE after LVAD implantation in comparison to pre‐LVAD examination (14 ± 3 vs. 12 ± 3 mm, *p* < 0.001).

The amount of MR improved by more than one grade during the first postoperative month (from 1.8 ± 1.0 to 0.8 ± 0.8, *p* < 0.001) and remained unchanged thereafter (0.9 ± 0.8 vs. 0.9 ± 0.8, *p* = 0.5). Similarly, the TR grade decreased considerably at the time of the first follow‐up compared with baseline measurements before LVAD (1.6 ± 1.0 vs. 1.2 ± 1, *p* < 0.001) but then remained unchanged (1.3 ± 1.0 vs. 1.2 ± 0.9, *p* = 0.1).

The improvement of MR and TR was found in both groups of patients with‐ and without previous valve surgery. However, patients with previous tricuspid surgery showed more frequently a significant TR improvement in comparison to patients without (*p* = 0.031). Among the patients with previous tricuspid surgery 40% experienced TR improvement by more than one grade compared with only 15% of patients without tricuspid surgery. Previous mitral valve surgery did not show any significant effect on MR post‐LVAD.

### 3.3 Prevalence and Echocardiographic Predictors of VAs

Overall, 102 patients (39%) had appropriate ICD interventions due to sustained VAs on LVAD support, for treatment of MVT in 100 patients, PVT/VF in 27 patients, and both in 19 patients (categories not mutually exclusive). We assessed the impact of the following pre‐LVAD echocardiographic parameters on the occurrence of post‐LVAD VAs: LVEDD, LVESD, LVEF, RVEDD, TAPSE, RV‐RA pressure gradient, MR and TR. The analysis was performed for all VAs together and separately for MVT and VF.

In the univariate analysis, pre‐LVEDD for all types of VA and both pre‐LVEDD and pre‐RVEDD for MVT predicted VA occurrence after LVAD implantation.

On multivariate analysis, pre‐LVEDD remained significantly predictive of VAs only in ICM patients, whereas pre‐RVEDD remained significantly predictive of VAs only for NICM patients, adjusted for age and sex.

**Figure 2:**
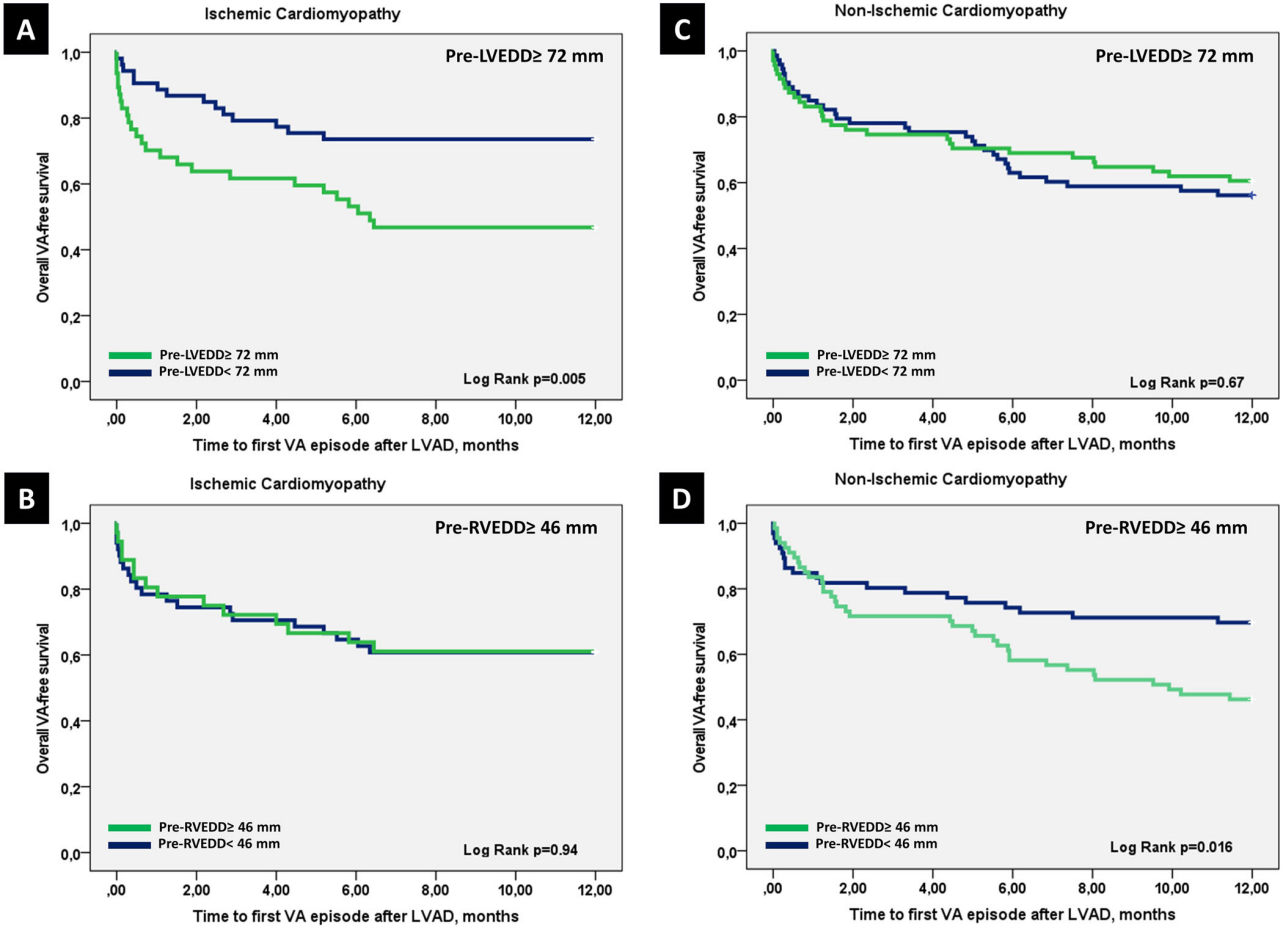
Kaplan–Meier plot of the time to the first VA event according to pre‐LVEDD in ICM patients (A), pre‐RVEDD in ICM patients (B), pre‐LVEDD in NICM patients (C), and pre‐RVEDD in NICM patients (D). (A) 8‐month rate for ICM patients with pre‐LVEDD ≥ 72 mm 47% CI [32.5–61.1], for patients with pre‐LVEDD < 72 mm 74% CI [61.6–85.6]. (B) 8‐month rate for ICM patients with pre‐RVEDD ≥ 46 mm 61% CI [45.2–77.0], for patients with pre‐RVEDD < 46 mm 61% CI [47.5–74.1]. (C) 8‐month rate for NICM patients with pre‐LVEDD ≥ 72 mm 68% CI [56.6–78.6], for patients with pre‐LVAD < 72 mm 59% CI [47.5–70.3]. (D) 8‐month rate for NICM patients with pre‐RVEDD ≥ 46 mm 55% CI [43.2–67.2], for patients with pre‐RVEDD < 46 mm 71% CI [60.2–82.2].

In the cox regression analysis, pre‐LVEDD ≥ 72 mm predicted the occurrence of VAs after LVAD implantation for ICM patients (HR: 2.9, confidence interval (CI): [1.3–6.6], *p* = 0.012), and pre‐RVEDD ≥ 46 mm was predictive for post‐LVAD VAs in NICM patients (HR: 2.8, (CI): [1.4–5.9], *p *= 0.004) (Figure 2A–D).

After including other known predictors such as atrial fibrillation and pre‐LVAD VAs in the analysis, a pre‐LVEDD ≥ 72 mm remained a significant predictor of VAs in patients with ICM (HR: 2.0, CI: [1.0–4.3], *p* = 0.04). In patients with NICM, pre‐LVAD VAs were the most significant predictor for post‐LVAD VAs. A pre‐RVEDD of ≥ 46 mm was linked to a 1.7‐fold increase in the risk of post‐LVAD VAs, although this association did not reach statistical significance, likely due to the small patient sample size.

**Figure 3:**
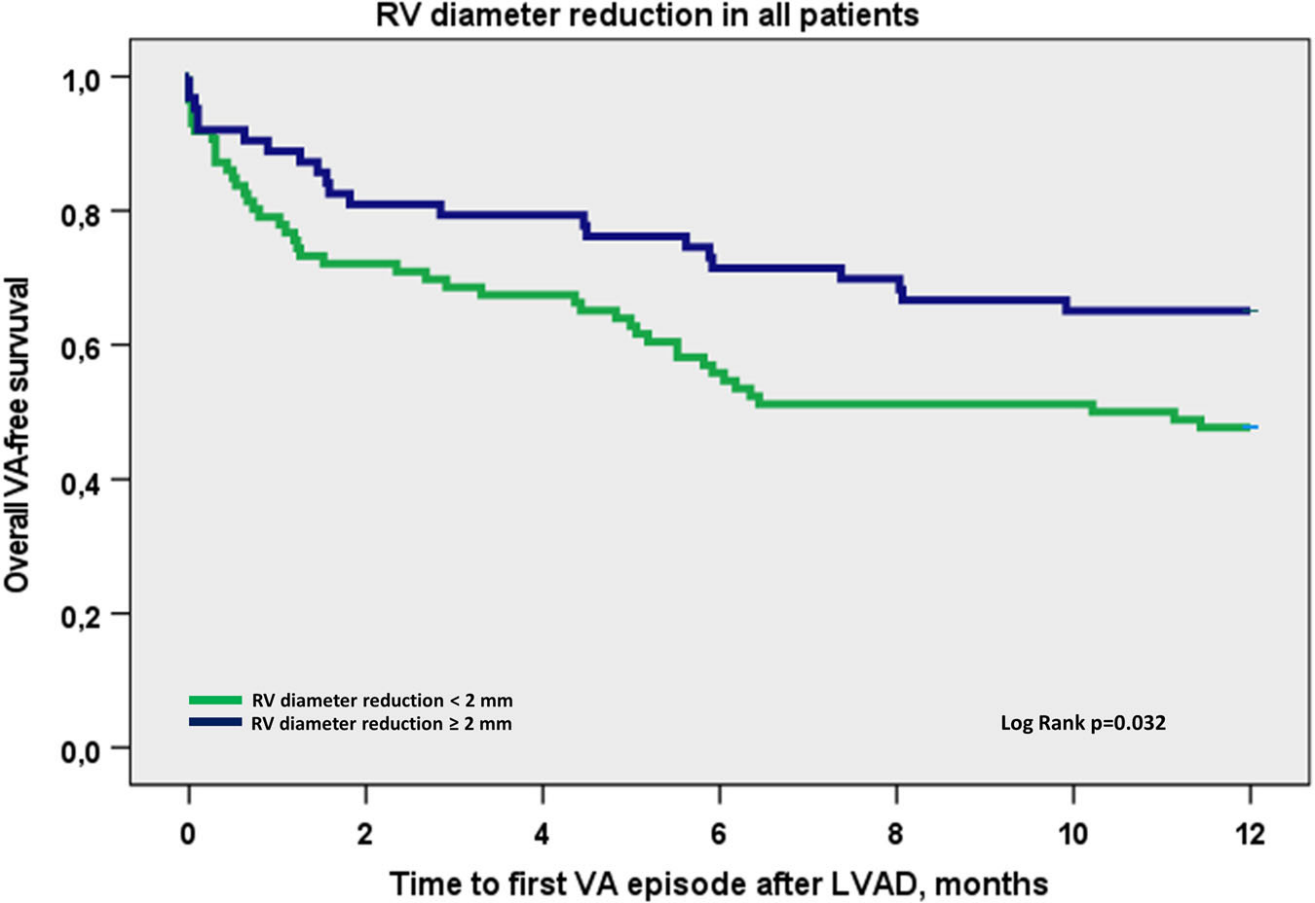
Kaplan–Meier plot of the time to the first VA event according to RV diameter reduction at 1 year after LVAD. 8‐month rate for patients with RV diameter reduction ≥ 2 mm 66% CI [54.3–77.9], for those with RV diameter reduction < 2 mm 51% CI [39.8–61.4].

A larger RVEDD at 1 year follow‐up, was highly associated with the VA occurrence in the first post‐LVAD year (50 ± 10 vs. 45 ± 8 mm, *p* = 0.001). Reduction of the RV diameter ≥ 2 mm at the 1‐year follow‐up was significantly associated with fewer VAs in the first postoperative year (*p* = 0.032) (Figure 3). All other obtained echocardiographic parameters were similar in patients with and without VAs.

At time of the first post‐LVAD echocardiography, 10 patients (10%) with and 19 (12%) without post‐LVAD VAs demonstrated a leftward shift of IVS. At 1‐year follow‐up, 12 patients from each group demonstrated a leftward shift of IVS. We detected overall nine “suction” events during follow‐up visits; but only in one patient (HVAD) was the LVAD “suction” chronologically related to VAs.

We compared the LVAD pump speed between patients with and without post‐LVAD VAs and did not find any association in all types of LVAD.

## 4 Discussion

To the best of our knowledge, this is the first study that has examined echocardiographic predictors for the occurrence of post‐LVAD VAs.

### 4.1 Main Findings

The occurrence rate of post‐LVAD VAs of 39% in our study is consistent with the previously reported one (range from 20% to 52%) [8, 9, 10, 11, 12, 13, 14]. The echocardiographic markers that were associated with post‐LVAD VAs were preoperative LVEDD and RVEDD, despite the effective unloading under LVAD.

The key finding was the pre‐LVEDD being predictive for post‐LVAD VAs in ICM patients exclusively, whereas the pre‐RVEDD was predictive for VAs in NICM patients solely.

It is known that cardiac chamber dilatation correlates well with the amount of arrhythmogenic substrate [15, 16]. More extensive preoperative substrate might be a predictor of higher risk for developing post‐LVAD VAs. Indeed, pre‐LVAD VAs have been shown to be a powerful predictor of post‐LVAD VAs [10, 17, 18], which reflects the presence of the preexisting arrhythmogenic substrate. Rosenbaum et al. [14] also demonstrated a pivotal role of the preoperative proarrhythmogenic substrate in the occurrence of post‐LVAD VAs. Among all treated post‐LVAD VAs, the shock‐terminated VAs increased in frequency from baseline and declined after 1 month whereas the occurrence of ATP‐terminated episodes remained increased throughout the 5‐months follow‐up period. Furthermore, the authors compared the prevalence of postoperative VAs between patients who received LVAD and those with non‐VAD cardiac surgery. LVAD patients showed significantly more of both: shock‐terminated episodes and monitored VAs in comparison to non‐VAD patients.

Thus, the underlying arrhythmogenic substrate seems to be a leading cause of post‐LVAD VAs. We found the predictive value of pre‐LVEDD for the occurrence of post‐LVAD VAs in ICM patients. It is reasonable as the post‐infarction myocardial scar is predominantly confined to the LV, while the RV is rarely involved in the post‐infarction tachycardia in patients with ICM [19]. So the dilated LV in ICM might be interpreted as a sign of advanced left ventricular remodeling with an underlying arrhythmogenic substrate.

More interesting is the finding of the predictive value of pre‐RVEDD for the occurrence of post‐LVAD VAs in NICM patients. The arrhythmogenic substrate in NICM differs from ICM and is related to myocardial fibrosis. Compared to ICM, fibrotic areas in NICM are rarely compact, typically patchy and/or diffuse, and often non‐transmural, as was seen in the whole heart histology [20]. Due to the heterogeneity of the NICM cohort, many patients with NICM present with smaller left ventricles compared to those with ICM. Consequently, the arrhythmogenic substrate in NICM does not necessarily impact LV size or function. Several studies have evaluated the impact of the arrhythmogenic substrate on the occurrence of VAs, morbidity, and SCD in patients with NICM. In the study of Piers et al. the presence of myocardial scar estimated with LGE predicted MVT, but not PVT/VF in patients with NIDCM [16].

In a meta‐analysis performed by Kuruvilla et al. late gadolinium enhancement in nonischemic cardiomyopathy patients was associated with increased risk of appropriate ICD therapies, sudden cardiac death, heart failure hospitalization rate, and all‐cause mortality [21].

A right ventricular dilatation in heart failure patients is a sign of the advanced disease stage with presumably a more extensive arrhythmogenic substrate. Even under the effective LV unloading a larger right ventricle during the first postoperative year was predictive of post‐LVAD VAs in all patients. It might be of clinical relevance as post‐LVAD VAs could possibly affect mortality. The correlation of post‐LVAD VAs with mortality was demonstrated by some authors [2, 22, 23, 24]. In a meta‐analysis from Makki et al., post‐LVAD VAs were associated with an increased risk of all‐cause mortality after adjusting for competing risk factors at 60, 120, and 180 days of follow‐up [2].

We did not find any impact of VAs on mortality in the first year after LVAD implantation. Our patient cohort was heterogeneous with three types of LVAD being implanted, predominantly HVAD. It might be that in the first post‐LVAD year such conditions as sepsis, thrombosis etc., rather than VAs lead to death. In a previous study from our group, the crucial effect of VAs on outcome emerged only after 1 year on LVAD support [18]. Thus, the evaluation of the morphological substrate with modern imaging modalities before LVAD implantation might be of a great importance as this might lead to more aggressive treatment of HF and to early management of VAs in this patient group.

### 4.2 LV Unloading and Its Impact on the VA Occurrence

Similar to previous studies [3, 25, 26, 27], we found a significant reduction in LVEDD and a significant improvement of MR as a correlate of effective LV unloading post‐LVAD. We did not see any significant differences in echocardiographic markers of LV unloading between patients with and without VAs.

### 4.3 TR Regurgitation and RV Function in LVAD Patients

Similar to the results of Topilsky et al., who found TR improvement only in patients with concurrent tricuspid valve intervention [5], we found a better improvement in TR under LVAD in patients with previous tricuspid valve surgery. Topilsky et al. also reported a significant improvement of RV function as estimated by the reduction of RV‐ and RA pressure. Similarly, our patient cohort showed a significant reduction of the RV‐RA pressure gradient on LVAD, but not an improvement of the TAPSE.

In another study by Chapman et al. in HeartMate II patients [26], the authors did not observe any significant changes in the RV diameter under LVAD support, consistent with our observations. In contrast to our patients, the degree of TR in the study of Chapman did not change at all under LVAD. Moreover, the authors described no significant changes in RV function assessed semi‐quantitatively.

TAPSE is a regional marker of RV function. However, there have been data suggesting that TAPSE could be reduced after cardiac surgery despite the presence of a normal global RV function secondary due to changes of RV contractility [28, 29]. Taking into account the improvement of the RV‐RA gradient in our patients, we postulate that the reduced TAPSE more likely is caused by the LVAD surgery itself rather than to a deterioration of the global RV function. Improvement of RV function after LVAD seems to be relevant in regard to post‐LVAD VAs. A larger RVEDD at 1 year after LVAD was highly associated with the occurrence of post‐LVAD VAs in our patients. Based on the results of our study, further research should be aimed at: (1) the assessment of prognostic benefit of ICD implantation in LVAD patients without ICD who has a LVEDD ≥ 72 mm for ICM and a RVEDD ≥ 46 mm for NICM (2) the determination of the underlying morphological substrate before LVAD surgery to stratify the postoperative arrhythmogenic risk (3) the evaluation of early and more aggressive treatment strategies of heart failure and VAs (4) the evaluation of early tricuspid surgery in patients with significant TR to preserve RV function.

## 5 Conclusions

Preoperative end‐diastolic left and right ventricular diameters predict the occurrence of post‐LVAD VAs in ICM and NICM patients, respectively. The presence and the extent of the morphological substrate before LVAD are likely to determine the occurrence of post‐LVAD VAs.

## 6 Limitations

This study was a retrospective analysis of echocardiography examinations, which are subject to inter‐ and intraobserver variability. To more precisely identify all ventricular events, we included in the analysis only patients with an ICD after LVAD. We cannot generalize our results to patients without an implanted ICD. We believe that some more LVAD patients might have any self‐terminated VAs that were left unrecognized under LVAD support.

Furthermore, ICD programming and tachyarrhythmia treatment settings were individualized for each patient so that the patients with more aggressive therapy settings might have shown a higher prevalence of VA events.

## Conflicts of Interest

The authors declare no conflicts of interest.

## Data Availability

The data that support the findings of this study are available on request from the corresponding author. The data are not publicly available due to privacy or ethical restrictions.
